# Type 1 diabetes in a patient with Ellis-van Creveld syndrome

**DOI:** 10.1590/S1516-31802012000100009

**Published:** 2012-02-13

**Authors:** Carla Graziadio, Pricila Bernardi, Rafael Fabiano Machado Rosa, Paulo Ricardo Gazzola Zen, Giorgio Adriano Paskulin

**Affiliations:** I MD. Assistant Professor and Clinical Geneticist, Universidade Federal de Ciências da Saúde de Porto Alegre (UFCSPA), and Complexo Hospitalar Santa Casa de Porto Alegre (CHSCPA), Porto Alegre, Rio Grande do Sul, Brazil.; II MD. Clinical Geneticist, Universidade Federal de Ciências da Saúde de Porto Alegre (UFCSPA), and Complexo Hospitalar Santa Casa de Porto Alegre (CHSCPA), Porto Alegre, Rio Grande do Sul, Brazil.; III PhD. Adjunct Professor of Clinical Genetics and Professor of the Postgraduate Program on Pathology and Clinical Genetics, Universidade Federal de Ciências da Saúde de Porto Alegre (UFCSPA), and Complexo Hospitalar Santa Casa de Porto Alegre (CHSCPA), Porto Alegre, Rio Grande do Sul, Brazil.; IV PhD. Associate Professor of Clinical Genetics and professor of the Postgraduate Program on Pathology, Clinical Genetics and Cytogenetics, Universidade Federal de Ciências da Saúde de Porto Alegre (UFCSPA), and Complexo Hospitalar Santa Casa de Porto Alegre (CHSCPA), Porto Alegre, Rio Grande do Sul, Brazil.

**Keywords:** Diabetes mellitus, Type 1, Ellis-Van Creveld syndrome, Polydactyly, Dwarfism, Consanguinity, Diabetes mellitus tipo 1, Síndrome de Ellis-van Creveld, Polidactilia, Nanismo, Consanguinidade

## Abstract

**CONTEXT:**

Ellis-van Creveld (EVC) syndrome is a rare autosomal recessive disease characterized by disproportionate short stature, narrow thorax, postaxial polydactyly, nail and tooth abnormalities and congenital heart disease.

**CASE REPORT:**

The patient was a 22-year-old Caucasian man, the third child of consanguineous parents. He received the diagnosis of insulin-dependent diabetes mellitus (DM) at 16 years of age, and around one year later, he underwent surgery to correct a partial atrioventricular septal defect. Upon physical examination, at 22 years of age, he presented stature of 145.5 cm (P3), weight of 49 kg (P3), head circumference of 54 cm (P2-50), high palate, absence of one of the lower lateral incisor teeth, narrow shoulders, narrowing of the upper thorax, scoliosis, rhizomelic shortening of the upper limbs, brachydactyly, postaxial polydactyly and clinodactyly of the second and third fingers. The lower limbs showed rhizomelic shortening with significant genu valgum (knock-knee deformity), small feet with postaxial polydactyly, syndactyly between the second and third toes and hallux valgus. Multiple melanocytic nevi were evident on the face, thorax and limbs. At that time, he was using neutral protamine Hagedorn (NPH) insulin, with poorly controlled DM. The clinical findings presented led to the diagnosis of EVC syndrome. Only one case of this syndrome has been described with DM so far. Attention is drawn to the fact that the genes associated with this syndrome are located close to those of the Wolfram syndrome, a condition that leads to early-onset diabetes.

## RESUMO

**CONTEXTO:**

A síndrome de Ellis-van Creveld (EVC) é uma doença autossômica recessiva rara, caracterizada por baixa estatura desproporcionada, tórax estreito, polidactilia pós-axial, anormalidades em unhas e dentes e cardiopatia congênita.

**RELATO DO CASO:**

O paciente é um rapaz caucasiano de 22 anos, o terceiro filho de pais consanguíneos. Recebeu diagnóstico de diabetes melito (DM) insulino-dependente aos 16 anos, sendo que, cerca de um ano depois, foi submetido a cirurgia cardíaca de correção de defeito de septo atrioventricular parcial. Ao exame físico, aos 22 anos, ele apresentava estatura de 145,5 cm (P3), peso de 49 kg (P3), perímetro cefálico de 54 cm (P2-50), palato alto, ausência de um dos dentes incisivos inferiores laterais, ombros estreitos, estreitamento do tórax superior, escoliose, encurtamento rizomélico dos membros superiores, braquidactilia, polidactilia pós-axial e clinodactilia dos segundo e terceiro dedos. Nos membros inferiores, observava-se encurtamento rizomélico com importante geno valgo (deformidade dos joelhos-batidos), pés pequenos com polidactlia pós-axial, sindactilia entre segundo e terceiro dedos, e háluces valgos. Múltiplos nevos melanocíticos eram evidentes na face, tórax e membros. Neste momento ele está em uso de insulina NPH (neutral protamine Hagedorn), com um controle inadequado do DM. Seus achados clínicos levaram ao diagnóstico de síndrome de EVC. Apenas um caso desta síndrome foi descrito com DM até o momento, sendo que chama a atenção o fato de que os genes associados à síndrome se localizam próximo ao da síndrome de Wolfram, uma condição que cursa com diabetes de início precoce.

## INTRODUCTION

Ellis-van Creveld (EVC) syndrome (OMIM 225500),^1^ also called chondro-ectodermal or mesodermal-ectodermal dysplasia, is a very rare genetic disease that displays an autosomal recessive trait.^2^^,^^3^ Its exact prevalence remains unknown, but more than 150 cases have so far been reported, with higher frequency among the Amish community.^3^ EVC syndrome is considered to be a heterogeneous genetic condition, in which loss-of-function mutations in the genes *EVC* and *EVC2* (both located at 4p16) are responsible for about half of the patients with EVC syndrome. The phenotype is considered indistinguishable among patients with mutations in these genes.^4^

EVC syndrome is one of the short-rib polydactyly syndromes characterized by disproportionately short stature, small chest, postaxial polydactyly, abnormalities in nails and teeth and congenital heart defect. However, inter and intra-familial variability has been reported. Mortality and morbidity in cases of this condition are frequently determined by pulmonary and cardiac complications and, additionally, some disability may result from knock-knee deformity. No other health problems are common in this syndrome.^2^^,^^3^^,^^4^

We report here a man presenting a very rare association between EVC syndrome and type 1 diabetes mellitus (DM). 

## CASE REPORT

**Figure 1.**
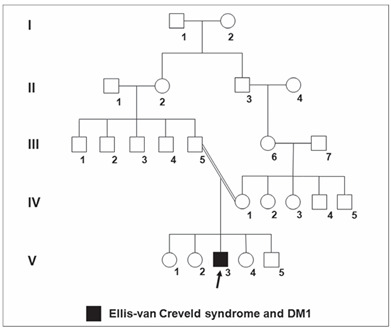
Pedigree of the patient’s family (subject V-3), showing the consanguinity observed between his parents (III-5 and IV-1) (DM1: type 1 diabetes mellitus).

The patient was a 22-year-old Caucasian man, the third child of a young, healthy consanguineous couple (the parents were first-degree cousins) (Figure 1), without similar cases in the family. He was born by means of vaginal delivery, at term, weighing 3,350 g (P25-50) and measuring 38 cm (P < 3). He did not present any intercurrence at birth, but limb abnormalities were identified. The pregnancy had been uneventful. 

The child evolved with a normal neuropsychomotor development: he could hold up his head at three months of age, sat up without support at five months, walked alone at 11 months and pronounced his first words at around one year of age. At two years of age, he was evaluated because of growth retardation, and at this stage, an endocrinologist indicated treatment with calcium and vitamin D. At three years of age, he began to develop genu valgum. At 12 years of age he underwent femoral varization osteotomy. He began school at seven years of age, continued as far as the fifth year, which he did twice, and then dropped out of school. Insulin-dependent diabetes was diagnosed at 16 years of age at a primary healthcare center, and around one year later, he underwent correction of a partial atrioventricular septal defect (AVSD).

**Figure 2.**
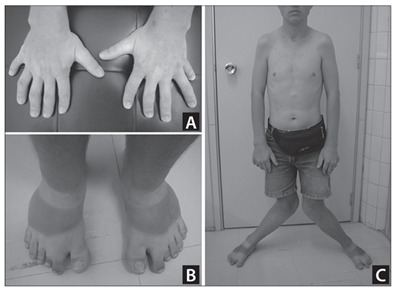
Patient’s appearance at the age of 22 years. Note especially the brachydactyly and polydactyly of the hands (A) and feet (B), several hypoplastic fingernails (A) and toenails (B), narrow shoulders and thorax, and the very significant genu valgum (knock-knee deformity) (C).

On physical examination, at 22 years of age, he presented height of 145.5 cm (P3), weight of 49 kg (P3), head circumference of 54 cm (P2-50), bulbous nasal point, high arched palate, absence of one of the lower lateral incisors, narrow shoulders, narrowing of the upper thorax, scoliosis, rhizomelic shortening of the upper limbs with cubitus valgus, brachydactyly (most pronounced in the third fingers, which also showed nails that were smaller than those of the other fingers), postaxial polydactyly, clinodactyly (ulnar deviation) of the second and third fingers and several hypoplastic nails. The lower limbs showed rhizomelic shortening with significant genu valgum (knock-knee deformity), small feet with postaxial polydactyly, small toes (especially the third toes) with several hypoplastic nails, syndactyly between the second and third toes, bilateral enlargement of the space between the first and second toes, and hallux valgus. Multiple melanocytic nevi were evident on his face, thorax and limbs (Figure 2). Multiple labial-gingival frenulae were not observed.

**Figure 3.**
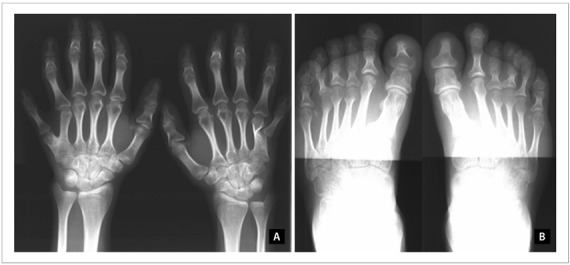
Radiographs on the patient’s hands and feet. Note especially the post-axial polydactyly, brachydactyly with short metacarpal and metatarsal bones and phalanges, and conical epiphyses (A and B).

Radiographic evaluation revealed dextroconvex thoracic scoliosis; rectification with inversion of the physiological kyphosis; brachyphalangia with shortening of the metacarpal and metatarsal bones of both the hands and the feet; postaxial polydactyly of the hands with duplication of the fifth metacarpal bones; conical epiphyses; significant bilateral genu valgum; curving of the tubular bones (most notably in the lower limbs) with deformity of the epiphyses; foot deformity with enlargement of the metatarsal bones and phalanges; postaxial polydactyly with six metatarsal bones in both feet; and presence of osteochondromas and osteoporosis (Figure 3).

He had symptoms of dysuria and mictional urgency, and a urodynamic evaluation revealed detrusor hyperactivity, a bladder with diminished capacity and complacency, diminished urinary flow and increased sensitivity suggestive of irritative and infectious factors, including diabetes. Abdominal ultrasound did not identify any presence of structural abnormalities. The patient also had a history of knee and back pain that had increased over the last few months. He was being followed up at the orthopedics department of the hospital because of the significant genu valgum. Furthermore, he was receiving care in the endocrinology department and was using NPH (neutral protamine Hagedorn) insulin, but with poor diabetes control. He presented glucose levels ranging between 180 and 314 mg/dl in the morning, and monitoring was being conducted at a primary healthcare center.

He started treatment in the hospital at the age of 22 years. NPH insulin was initially prescribed, consisting of 25 units before breakfast and 10 units before dinner. However, his lack of diabetes control persisted, and he showed symptoms of polyuria and nocturia. His blood glucose levels before breakfast ranged from 194 to 333 mg/dl; before lunch from 179 to 27 mg/dl and before dinner from 99 to 222 mg/dl. His glycated hemoglobin level was 8.7% (normal values range from 3.9 to 6.1%). Subsequently, the NPH insulin dose was changed to 26 units before breakfast and 14 units before dinner. After this, the patient developed episodes of hypoglycemia. His blood glucose levels before breakfast ranged from 153 to 225 mg/dl, before lunch from 51 to 282 mg/dl and before dinner from 54 to 229 mg/dl. The new glycated hemoglobin level was 9.5%, while the qualitative examination of urine showed a cross (+) for the presence of glucose. In the latest assessment, the scheme was changed to an NPH dose of 28 units before breakfast and 18 units before dinner. 

An ophthalmological evaluation showed the presence of cataracts, with an indication for surgery. His blood pressure was normal (mean of 120 x 80 mmHg). Thyroid, liver and kidney function tests were all normal. The patient was also reevaluated in the cardiology department and underwent an electrocardiogram, which was normal.

**Table 1.** Review of medical databases using the descriptors corresponding to the main features presented by the patient, conducted on December 13, 2010

Data base	Strategy of search	Results*
PubMed	“Ellis Van Creveld Syndrome” AND “Diabetes Mellitus”	1 case report
		1 article
		0 reviews
Scirus	“Ellis Van Creveld Syndrome” AND “Diabetes Mellitus”	60 articles
Embase	“Ellis Van Creveld Syndrome” AND “Diabetes Mellitus”	0 case reports
		2 reviews

*There were no results from using the same search strategies in the Cochrane Library, SciELO and Lilacs databases.

## DISCUSSION

EVC syndrome is considered to be a condition with variable features that involve multiple organs. The findings observed in our patient, of disproportionately short stature, polydactyly of the hands and feet, nail and tooth abnormalities and cardiac malformation, supported this diagnosis. The prognosis in cases of EVC syndrome is commonly determined by cardiopulmonary problems, especially during the neonatal period. Congenital heart defects have been described in up to 60% of the patients with EVC syndrome, and atrioventricular canal defects are the most frequently observed malformations,^3^^,^^4^ as seen in our patient. Other congenital heart malformations that have been described include a single atrium, defects of the mitral and tricuspid valves, patent ductus, ventricular septal defect, atrial septal defect and hypoplastic left heart syndrome.^3^^,^^4^

Diabetes mellitus is not considered to be a component of EVC syndrome, and, in our literature review, using the PubMed, Scirus, Embase, Cochrane Library, Lilacs and SciElo databases, we saw only one report describing this association^5^ (Table 1). About half of the cases of EVC syndrome are determined by *EVC* and *EVC2* gene mutations, and these genes are located in the 4p16 region.^4^ We draw attention to the fact that the *WFS1* gene is also located in this region and determines the Wolfram syndrome (WS, OMIM 2223000),^1^ which is an autosomal recessive condition also referred to as DIDMOAD (diabetes insipidus, diabetes mellitus, optic atrophy and deafness).^6^^,^^7^ Occurrences of diabetes mellitus and optic atrophy together is sufficient for diagnosing WS.^6^^,^^7^ However, until the latest assessment of our patient, at the age of 22 years, only the DM was observed, which would be not expected for a patient with WS (usually, patients with WS present optic atrophy before the age of 19 years).^7^

Autoimmune conditions are uncommon among patients with EVC syndrome.^2^^,^^3^^,^^4^ Type 1 DM is responsible for about 5-10% of all cases of diabetes, and susceptibility to this is considered to be largely inherited, residing especially in the HLA genotypes DR and DQ.^8^ However, several different candidate genes have been proposed,^8^ and a relationship between type 1 DM and the consanguinity observed in our patient cannot be ruled out.

Thus, more reports will be necessary in order to determine whether the type 1 DM observed in our patient may be a true feature of the clinical spectrum of the syndrome. We cannot rule out the possibility that the association between EVC syndrome and DM observed in our patient may have been merely coincidental.
